# Effect of direct mailing self-sampling kits on participation in the Dutch hrHPV-based cervical screening programme: a population-based study

**DOI:** 10.1016/j.lanepe.2026.101635

**Published:** 2026-03-12

**Authors:** Ellen M.G. Olthof, Albert G. Siebers, Inge M.C.M. de Kok

**Affiliations:** a Erasmus MC University Medical Center Rotterdam, Department of Public Health, Rotterdam, the Netherlands; b The Dutch Nationwide Pathology Databank (Palga Foundation), Houten, the Netherlands

**Keywords:** Self-sampling, Participation, Direct mailing, Cervical cancer screening, HPV screening

## Abstract

**Background:**

In July 2023, the Dutch cervical cancer screening programme introduced direct mailing of self-sampling kits with invitations for 30-year-olds and with reminder for non-participants in other age groups after 12 weeks. This study evaluates the impact of this new policy on participation, self-sampling uptake, and follow-up testing.

**Methods:**

Data from the national screening (ScreenIT) and pathology (Palga) databases were used. Individuals invited between July 11 and December 31, 2023, were compared to a control group from the same period in 2022. Primary outcomes included participation rates following invitation and reminder, time to participation, self-sampling uptake, reflex cytology participation among high-risk HPV(hrHPV)-positive self-sampling participants, and first-time participation. Analyses were stratified by age and screening history.

**Findings:**

Overall participation increased from 42.1% (n = 330,413) in the control group to 49.9% (n = 327,376; RR: 1.19, 95% CI: 1.18–1.19) with the new policy. After primary invitation, the largest increase in participation from 30.6% to 40.3% was observed among 30-year-olds (RR: 1.32, 95% CI: 1.30–1.34). Vaginal self-sampling uptake increased from 21.9% to 61.9% (RR: 2.82, 95% CI: 2.79–2.85), with the largest uptake among first-time participants (previous non-attenders, 32.1%–78.1%; RR: 2.43, 95% CI: 2.35–2.51) and 30–34-year-olds (25.1%–83.7%; RR: 3.33, 95% CI: 2.25–3.40). The proportion of first-time participants increased from 6.8% in the control group to 9.1% with the new policy. Participation increased sharply in individuals aged 35+ after 12 weeks. Reflex cytology participation among hrHPV-positive self-sampling participants declined by 6% (from 93% to 87%; RR: 0.94, 95% CI: 0.93–0.95), but this did not affect the overall increase in participation rates.

**Interpretation:**

Direct mailing of self-sampling kits is an effective strategy to increase participation in population-based cervical cancer screening programmes, especially among first-time invitees and previous non-attenders. Continued participation in reflex cytology among hrHPV-positive women remains essential to maximise the overall impact of the policy on screening effectiveness.

**Funding:**

This study was funded by the National Institute for Public Health and the Environment.

**Research in context**

**Evidence before this study**

Participation rates are declining in population-based cancer screening programmes in the Netherlands. Evidence has shown that self-sampling can increase participation by reducing barriers associated with traditional smear tests performed by a GP. We searched PubMed for peer-reviewed papers published from inception up until 01/06/2025 using the search terms self-sampling AND participation AND cervical screening. We excluded studies that focused on populations outside of Europe. Studies were included if they evaluated self-sampling for cervical screening in Europe and reported on participation rates. Studies using controlled settings, such as randomised controlled trials, have demonstrated that self-sampling increases participation in cervical cancer screening programmes, particularly when self-sampling kits are directly mailed to participants rather than requiring an opt-in request. However, evidence from real-world nationwide implementation in population-level screening programmes is currently lacking.

**Added value of this study**

This study is the first to evaluate the large-scale implementation of directly mailing self-sampling kits within a national, population-based cervical cancer screening programme. The Dutch programme is the first worldwide to incorporate direct mailing of self-sampling kits both with the initial invitation to 30-year-olds and with the reminder for other eligible age groups. Our findings demonstrate that this approach increases total participation by 8%, with a particularly high uptake among first-time participants and younger women. The results provide robust evidence of the feasibility and effectiveness of direct mailing self-sampling kits in a real-world setting, offering valuable insights into its impact on participation and the potential to enhance screening coverage on a population level.

**Implications of all the available evidence**

The direct mailing of self-sampling kits is an effective strategy to increase participation in organised cervical cancer screening programmes by lowering barriers to participation. This approach has the potential to improve overall screening coverage and reach underserved populations.

## Introduction

Since the introduction of primary high-risk human papillomavirus (hrHPV)-based screening in the Netherlands in 2017, participation rates have declined.^1^ The lowest participation rates are observed in the women first invited for screening at age 30.^2^ Research has shown that part of this decline can be attributed to changes in the invitation policy.^3^ However, most contributing factors remain unclear. Studies focussing on increasing participation have highlighted the effectiveness of direct mailing self-sampling kits as a strategy to reduce barriers to screening.^4^, ^5^, ^6^ A recent randomised clinical trial in the U.S. demonstrated that direct mailing self-sampling kits significantly increased participation rates, compared to the usual care (patient reminders) with educational materials, regardless of their screening history.^7^ Also the Dutch IMPROVE trial demonstrated that the majority of women (76.5%) preferred self-sampling compared to 11.9% who preferred clinician-based sampling.^8^

With the introduction of hrHPV-based screening in the Netherlands, self-sampling became an option for women who preferred not to participate with the traditional smear test taken by their GP. Women could request a self-sampling kit (opt-in), but uptake remained low at around 8%.^1^ During the COVID-19 pandemic, self-sampling was presented as equivalent to the traditional smear test to address delays in participation, which led to an increase in self-sampling uptake to 25% in 2021.^2^^,^^9^

Since July 3, 2023, the policy regarding self-sampling in the Dutch cervical cancer screening programme has been revised.^10^ Now, self-sampling kits are automatically sent with invitations for all individuals at age 30, and with the reminder to other eligible age groups if they have not participated within 12 weeks. The Netherlands is the first country that implemented direct mailing of self-sampling kits in a nationwide, organised cervical cancer screening programme (i.e., opt-out). Furthermore, self-sampling is now presented as equivalent to the traditional smear test in both the invitation letter and informational leaflet, rather than as an alternative for those unwilling to undergo a smear test. These changes are expected to enhance participation in screening.

If hrHPV is detected with self-sampling, participants are still required to visit their GP for a smear test for cytological examination (i.e. “reflex cytology”). Therefore, participation in reflex cytology remains essential.

The aim of this broader implementation of self-sampling is to improve accessibility within the population-based screening programme. This study evaluates the impact of the new self-sampling policy on participation rates following invitation and reminder in primary screening, time to participation, self-sampling uptake, and follow-up testing in the Dutch organised cervical cancer screening programme. Analyses will be stratified by age and screening history (first-time participants vs. previously screened participants).

## Methods

### Data selection

Data from the Dutch nationwide pathology databank (Palga) and from the national screening organisation (ScreenIT) was used. Individuals invited between July 11, 2023, and December 31, 2023 were included in the study. Follow-up for participation data continued until March 31, 2024. The control group consist of individuals invited between July 11, 2022 and December 31, 2022, with the same follow-up duration for participation (until March 31, 2023).

### Dutch cervical cancer screening programme

Women aged 30–65 are invited to participate in the Dutch cervical screening programme every five years. At ages 45 and 55, women are invited only if they were hrHPV-positive (and were not referred for colposcopy) or did not participate in the previous round of screening. Women aged 65 are invited only if they were hrHPV-positive at age 60 and have not been seen by a gynaecologist. The initial screening includes either self-sampling or a smear test collected at the General Practitioner (GP) practice, typically by a trained assistant or the GP. If hrHPV is detected, a cytological examination (i.e. cytology) is performed on the GP-collected sample. Women who use self-sampling are required to visit their GP for a smear test, as cytological examinations cannot be performed on self-sampled material.

Women with HPV16 or HPV18 and cytological abnormalities (Atypical Squamous Cells of Undetermined Significance or more severe, ASC-US+) are referred to a gynaecologist for colposcopy. Women with other hrHPV types showing high-grade squamous intraepithelial lesions (HSIL) are also referred for colposcopy. hrHPV-positive women without cytological abnormalities (NILM) or ASC-US/LSIL are cytologically retested with a control smear test after twelve months. If abnormalities (ASC-US+) are found in the repeat cytology, these women will be referred for colposcopy. If no cytological abnormalities are detected, women return to the regular screening programme after five years. HrHPV-negative women are referred back to the screening programme after five or ten years (depending on their age).

### Invitation and reminder policy before and after July 2023

Before July 2023, all eligible women received a primary invitation letter at the recommended age-specific intervals (every 5 years from age 30, with exceptions at ages 45, 55, and 65 depending on previous results and participation history). This invitation did not include a self-sampling kit. If women did not respond, one reminder letter was sent after 12 weeks, again without a kit. Women could request a self-sampling kit after either primary invitation or reminder. If they did not attend after reminder, no further follow-up was provided until the next screening round.

Since July 2023, the policy has been revised. At age 30, the primary invitation now includes a self-sampling kit, presented as an equivalent option to the smear test. If a woman does not respond to this invitation a reminder letter is sent, but without a (second) kit. For women in other eligible age groups, the primary invitation continues to be a letter without a kit, although women may request one. If no participation occurs within 12 weeks, a reminder letter is sent that now includes a self-sampling kit.

The reminder is sent 12 weeks after the primary invitation as part of the standard national screening protocol. Women remain eligible to participate at any time until the next invitation cycle. During the study period, access to GPs and to self-sampling kits was stable, with no known structural barriers affecting test uptake.

### Outcome indicators

The outcome indicators include: participation rates following invitations and reminders, the time between invitation and participation (i.e., cumulative response time; in weeks), participation in reflex cytology (for hrHPV-positive self-sampling participants) and the chosen sampling method (self-sampling vs. smear test). Participation rates were calculated as the total number of women screened within 15 months of the start of the year divided by the total number of invitations sent in that calendar year. Cumulative response time was calculated as the proportion of all invitees who had participated in screening at each week after invitation.

### Other indicators

Screening history was defined as individuals screened since 2008 and categorised into first-time participants (previous non-attenders) and previously screened participants. Age categories included 30–34, 35–39, 40–44, 45–49, 50–54, 55–59, 60–64, and 65–69 years.

### Data analysis

Overall participation rates, self-sampling uptake and participation rates in reflex cytology were calculated for each age category and compared between the new self-sampling policy and the control group. Participation rates reported after primary invitation and reminder were calculated using all invitees as the denominator. Self-sampling uptake was defined as the proportion of participants who chose self-sampling, with all screening participants as the denominator. Participation rates in reflex cytology were calculated using all hrHPV-positive self-sampling participants as the denominator. Differences in participation rates and self-sampling uptake between the new self-sampling policy and the control group were analysed by calculating relative risks with 95% confidence intervals. Analyses were stratified by age and screening history. For the analysis based on screening history, first-time invitees (i.e., individuals aged 30–34) were excluded. All analyses were conducted using IBM SPSS Statistics for Windows Version 25.0 (Armonk, NY: IBM Corp).

In addition, we calculated post-hoc adjusted overall participation to account for the decline in reflex cytology among hrHPV-positive self-sampling participants. Adjusted overall participation was defined as the proportion of invitees who either completed primary screening or, if hrHPV-positive, completed reflex cytology.

### Role of the funding source

This study was funded by the Dutch National Institute for Public Health and the Environment (Rijksinstituut voor Volksgezondheid en Milieu). The funding source was not involved in the study design, data collection, data analysis, interpretation of the data, writing of the report or the decision to submit the paper for publication.

## Results

### Participation rates after invitation and reminder

A total of 330,413 and 327,376 women were invited for screening in the control group and in the new-self sampling group, respectively. The implementation of the new self-sampling policy resulted in a significant increase in overall participation rates, rising from 42.1% during the control period to 49.9% (RR: 1.19, 95% CI: 1.18–1.19) under the new policy (Table 1). Participation rates after primary invitation increased most in women aged 30, with an increase from 30.6% to 40.3% (RR: 1.32, 95% CI: 1.30–1.34), after the inclusion of a self-sampling kit in the invitation. Additionally, overall participation rates following the reminder (with included self-sampling kit) increased from 4.9% to 11.5% (RR: 2.37, 95% CI: 2.33–2.41) with the new self-sampling policy (out of all invitees for screening, not just those who did not respond). This trend was observed consistently across all age groups.

**Table 1:** Overall participation rates and participation rates after primary invitation and reminder.

Age group	Control group (n = 330,413)	New SS group (n = 327,376)	Absolute difference	RR	95% CI
Overall particpation rates^a^ (denominator: all invitees)					
30–34	37.3%	48.4%	11.1%	1.30	**1.28–1.31**
35–39	39.7%	47.0%	7.3%	1.19	**1.17–1.20**
40–44	44.6%	51.6%	7.0%	1.16	**1.14–1.17**
45–49	23.3%	31.1%	7.8%	1.33	**1.29–1.37**
50–54	49.5%	55.5%	6.0%	1.12	**1.11–1.13**
55–59	24.7%	31.4%	6.7%	1.27	**1.23–1.31**
60–64	54.7%	62.4%	7.7%	1.14	**1.13–1.15**
65–69	78.0%	76.5%	−1.5%	0.98	0.94–1.03
Total	42.1%	49.9%	7.8%	1.19	**1.18–1.19**
Participation rates after primary invitation (denominator: all invitees)					
30–34	30.6%	40.3%	9.7%	1.32	**1.30–1.34**
35–39	32.6%	32.8%	0.2%	1.01	0.99–1.02
40–44	36.7%	36.7%	0.0%	1.00	0.99–1.02
45–49	18.4%	18.5%	0.1%	1.00	0.97–1.04
50–54	41.2%	40.4%	−0.8%	0.98	**0.97–1.00**
55–59	20.3%	20.1%	−0.2%	0.99	0.95–1.03
60–64	47.9%	49.7%	1.8%	1.04	**1.03–1.05**
65–69	70.9%	59.4%	−11.5%	0.84	**0.79–0.89**
Total	35.1%	37.2%	2.1%	1.06	**1.05–1.07**
Participation rates after reminder^b^ (denominator: all invitees)					
30–34	4.3%	7.7%	3.4%	1.78	**1.70–1.86**
35–39	4.6%	12.7%	8.1%	2.75	**2.64–2.87**
40–44	5.6%	13.3%	7.7%	2.39	**2.29–2.49**
45–49	3.5%	11.7%	8.2%	3.37	**3.12–3.64**
50–54	6.1%	13.5%	7.4%	2.19	**2.11–2.28**
55–59	3.0%	10.3%	7.3%	3.44	**3.16–3.75**
60–64	5.1%	11.5%	6.4%	2.27	**2.17–2.37**
65–69	5.8%	14.7%	8.9%	2.56	**1.91–3.43**
Total	4.9%	11.5%	6.6%	2.37	**2.33–2.41**

SS: self-sampling. The self-sampling kit was automatically sent with the invitation to the 30-year-olds and with the reminder to other eligible age groups. RR: Relative risk. CI: Confidence interval.

Significant results are indicated in bold (p < .005).

a Participation rates after primary invitation and reminder do not sum to overall participation rates, as participation after requesting a self-sampling kit is also included in the overall participation rates. Overall participation rates increased by 2.1% before the new self-sampling policy and by 1.2% after implementation after requesting a kit (data not shown).

b Participation rates after the reminder were calculated using the total population invited as the denominator, not just those who did not respond to the primary invitation.

### Response time between invitation and participation

**Fig. 1:**
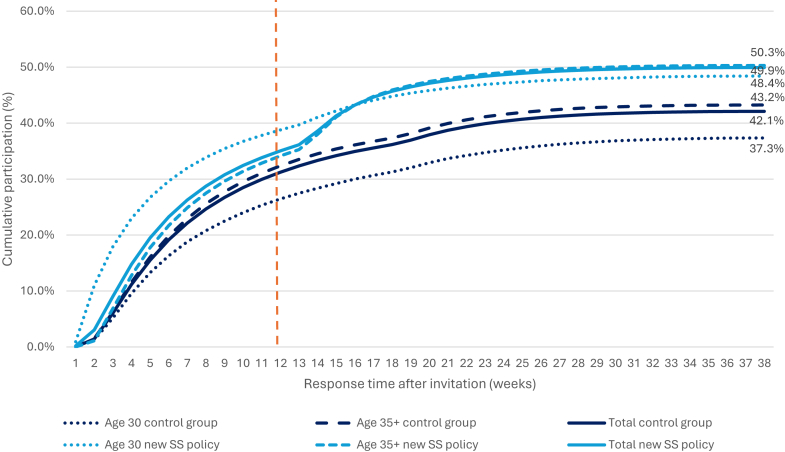
Cumulative participation over time after invitation (in weeks), calculated as the percentage of all invitees who had participated by each week following invitation. The self-sampling kit was automatically sent with the invitation to the 30-year-olds and with the reminder (after 12 weeks, indicated with the orange dotted line) to other eligible age groups (35+). SS: self-sampling.

Following the primary invitation, the most rapid increase in participation rates was observed in women aged 30 after the implementation of the new self-sampling policy, compared to the control group (Fig. 1). Furthermore, in the 35+ age group, a sharp increase in participation rates was observed starting 12 weeks after the primary invitation. This period coincided with the automatic inclusion of a self-sampling kit in the reminder. The average response time was 62.4 days in the control group and 60 days after the new self-sampling policy.

### Self-sampling uptake according to age

Participation through self-sampling significantly increased overall by 40 percentage points, from 21.9% to 61.9% (RR: 2.82, 95% CI: 2.79–2.85), and this trend was observed across all age categories after the introduction of the new self-sampling policy, compared to the control period (Table 2). The largest increase was observed in the 30–34 age group, where the use of self-sampling increased from 25.1% to 83.7% (RR: 3.33, 95% CI: 3.25–3.40). Other age groups used self-sampling ranging from 45.2% to 65.0% (with the new self-sampling policy), compared to a range of 12.3%–24.9% in the control group. The lowest usage of self-sampling (45.2%) after the new self-sampling policy was observed in the 65–69 age group.

**Table 2:** Self-sampling uptake by screening history and age after the introduction of the new self-sampling policy compared to the control group.

Age group	Control group (n = 139,104)	New SS group (n = 163,454)	Absolute difference	RR	95% CI
Overall proportion self-sampling uptake (denominator: all screening participants)					
30–34	25.1%	83.7%	58.6%	3.33	**3.25–3.40**
35–39	23.3%	57.1%	33.8%	2.45	**2.39–2.51**
40–44	20.2%	54.5%	34.3%	2.69	**2.62–2.77**
45–49	19.8%	60.5%	40.7%	3.05	**2.88–3.23**
50–54	19.8%	54.2%	34.4%	2.74	**2.67–2.81**
55–59	24.9%	65.0%	40.1%	2.62	**2.49–2.75**
60–64	21.8%	58.5%	36.7%	2.69	**2.62–2.75**
65–69	12.3%	45.2%	32.9%	3.67	**2.98–4.51**
Total	21.9%	61.9%	40.0%	2.82	**2.79–2.85**

Age group	Control group (n = 7540)	New SS group (n = 11,765)	Absolute difference	RR	95% CI
Proportion self-sampling uptake by first-time participants (denominator: all first-time participants)^a^					
30–34	*N/A*	*N/A*	*N/A*	*N/A*	*N/A*
35–39	29.5%	76.2%	46.7%	2.59	**2.44–2.74**
40–44	29.2%	77.2%	48.0%	2.64	**2.44–2.85**
45–49	28.0%	77.1%	49.1%	2.76	**2.48–3.06**
50–54	35.9%	79.2%	43.3%	2.20	**2.01–2.41**
55–59	41.8%	85.1%	43.3%	2.04	**1.86–2.23**
60–64	44.9%	83.7%	38.8%	1.86	**1.68–2.06**
65–69	–	–	–	–	**–**
Total	32.1%	78.1%	46.0%	2.43	**2.35–2.51**

Age group	Control group (n = 106,634)	New SS group (n = 117,052)	Absolute difference	RR	95% CI
Proportion self-sampling uptake by previously screened participants (denominator: all previously screened participants)^a^					
30–34	*N/A*	*N/A*	*N/A*	*N/A*	*N/A*
35–39	22.3%	55.3%	33.0%	2.48	**2.41–2.55**
40–44	19.5%	53.4%	33.9%	2.74	**2.66–2.82**
45–49	18.0%	58.7%	40.7%	3.27	**3.05–3.49**
50–54	19.1%	54.1%	35.0%	2.83	**2.75–2.91**
55–59	22.3%	64.3%	42.0%	2.89	**2.73–3.06**
60–64	21.3%	58.6%	37.3%	2.75	**2.69–2.82**
65–69	12.4%	52.8%	40.4%	4.26	**3.47–5.23**
Total	20.4%	56.1%	35.7%	2.75	**2.71–2.78**

SS: self-sampling. The self-sampling kit was automatically sent with the invitation to the 30-year-olds and with the reminder to other eligible age groups. RR: Relative risk. CI: Confidence Interval.

Significant results are indicated in bold (p < .005).

a Includes only participants for whom screening history was available.

### Self-sampling uptake according to screening history

A significant increase in the use of self-sampling was observed among both first-time participants and previously screened participants (Table 2). Among individuals who participated in screening for the first time, the overall use of self-sampling increased with 46.0 percentage points from 32.1% to 78.1% (RR: 2^.^43, 95% CI: 2.35–2.51) following the introduction of the new self-sampling policy, compared to the control group. Among participants who had previously been screened, the use of self-sampling increased with 35.7 percentage points from 20.4% to 56.1% (RR: 2.75, 95% CI: 2.71–2.78). This trend was consistent across all age groups.

### Participation in reflex-cytology after an hrHPV-positive self-sampling test

The participation in reflex-cytology following hrHPV-positive self-sampling has decreased with −5.7 percentage points (RR: 0.94, 95% CI: 0.93–0.95) after the new self-sampling policy compared to the control period (Table 3). The largest reduction from 84.1% to 70.8% (−13.3 percentage points; RR: 0.84, 95% CI: 0.79–0.90) in reflex cytology participation is observed among first-time participants. The declining trend is also observed among participants who have been previously screened, with reflex-cytology participation decreasing from 92.3% to 88.5% (−3.8 percentage points; RR: 0.96, 95% CI: 0.94–0.98).

**Table 3:** Participation in reflex cytology after hrHPV-positive self-sampling, by screening history and age after the introduction of the new self-sampling policy compared to the control group.

Age group	Control group (n = 2802)	New SS group (n = 14,837)	Absolute difference	RR	95% CI
Overall participation in reflex cytology (denominator: all hrHPV-positive SS participants)					
30–34	95.3%	88.9%	−6.4%	0.93	**0.92–0.95**
35–39	92.5%	84.2%	−8.3%	0.91	**0.89–0.94**
40–44	88.4%	85.6%	−2.8%	0.97	0.93–1.01
45–49	82.4%	83.6%	1.2%	1.02	0.94–1.10
50–54	94.3%	87.5%	−6.8%	0.93	**0.90–0.96**
55–59	90.3%	80.2%	−10.1%	0.89	**0.83–0.95**
60–64	93.4%	90.2%	−3.2%	0.97	0.93–1.00
65–69	94.1%	88.2%	−5.9%	0.94	0.85–1.03
Total	92.6%	86.9%	−5.7%	0.94	**0.93–0.95**

Age group	Control group (n = 264)	New SS group (n = 1350)	Absolute difference	RR	95% CI
Participation in reflex cytology by first-time participants (denominator: all hrHPV-positive SS first-time participants)^a^					
30–34	*N/A*	*N/A*	*N/A*	*N/A*	*N/A*
35–39	89.6%	74.0%	−15.6%	0.83	**0.76–0.89**
40–44	80.7%	70.2%	−10.5%	0.87	0.75–1.01
45–49	63.3%	74.1%	10.8%	1.17	0.88–1.56
50–54	93.3%	62.3%	−31.0%	0.67	**0.56–0.79**
55–59	80.0%	66.1%	−13.9%	0.83	0.64–1.07
60–64	83.3%	62.0%	−21.3%	0.74	0.54–1.02
65–69	–	–	–	–	–
Total	84.1%	70.8%	−13.3%	0.84	**0.79–0.90**

Age group	Control group (n = 1500)	New SS group (n = 7981)	Absolute difference	RR	95% CI
Participation in reflex cytology by previously screened participants (denominator: all hrHPV-positive SS previously screened participants)^a^					
30–34	*N/A*	*N/A*	*N/A*	*N/A*	*N/A*
35–39	93.4%	87.6%	−5.8%	0.94	**0.91–0.97**
40–44	89.7%	88.9%	−0.8%	0.99	0.95–1.04
45–49	87.3%	86.7%	−0.6%	0.99	0.92–1.07
50–54	94.4%	89.8%	−4.6%	0.95	**0.92–0.98**
55–59	92.3%	82.6%	−9.7%	0.90	**0.84–0.96**
60–64	93.9%	91.6%	−2.3%	0.98	0.94–1.01
65–69	94.1%	88.5%	−5.6%	0.94	0.86–1.03
Total	92.3%	88.5%	−3.8%	0.96	**0.94–0.98**

SS: self-sampling. The self-sampling kit was automatically sent with the invitation to the 30-year-olds and with the reminder to other eligible age groups. RR: Relative risk. CI: Confidence Interval.

Significant results are indicated in bold (p < .005).

a Includes only participants for whom screening history was available.

### Proportion of first-time participants on overall attendance rates

**Fig. 2:**
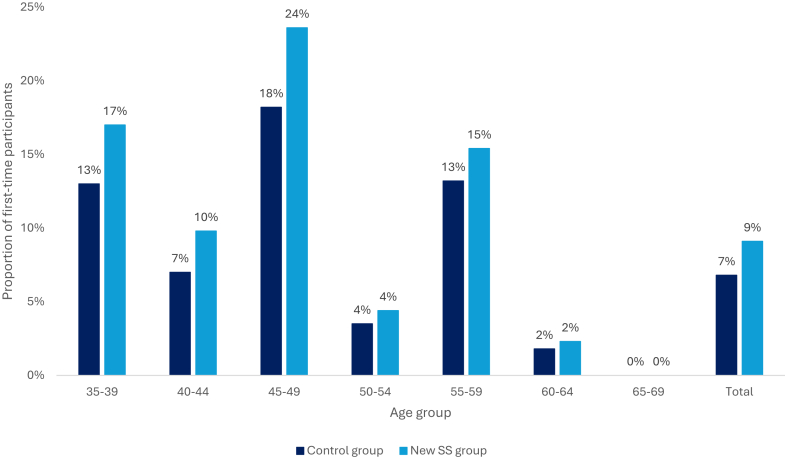
The proportion of first-time participants out of the total number of participants after the introduction of the new self-sampling policy compared to the control group. SS: self-sampling.

The proportion of first-time participants has increased from 7% to 9% after the new self-sampling policy compared to the control period (Fig. 2). The largest increase (from 18% to 24%) was observed in age group 45–49.

## Discussion

This study is the first to evaluate the implementation of directly mailing self-sampling kits in an organised cervical cancer screening programme. The direct mailing of self-sampling kits, both with the invitation and the reminder, led to a significant increase in participation rates and a faster response to the screening. The use of self-sampling notably increased across all age groups, with the most substantial increase observed in the 30–34 age group, who received a self-sampling kit with their initial invitation. Additionally, there was a marked increase in first-time participants (excluding the 30–34 age group) following the introduction of the new self-sampling policy, who predominantly used self-sampling. However, reflex-cytology participation after an hrHPV-positive self-sampling test showed a slight decrease across most age groups. Overall, the direct mailing of self-sampling kits seems to be an effective strategy for reducing barriers to participation in population-based screening programmes.

The observed decline in reflex cytology participation following an hrHPV-positive self-sampling test is noteworthy, especially among first-time participants. This suggests that while the introduction of self-sampling may lower the threshold for participation in cervical cancer screening, the subsequent step of undergoing a cervical smear at the GP still presents a barrier. Psychosocial factors, such as fear of the smear test, embarrassment, fear of test results, or a lack of understanding about the importance of follow-up testing, have been reported in systematic reviews as possible contributors to cervical screening uptake and may play a role in this observed decline.^11^ In addition, part of the decline may be explained by methodological factors. After introduction of the new self-sampling policy, the proportion of women who participated after the reminder doubled compared with the control group. Because self-sampling kits were mailed with the reminder, these women had approximately 12 weeks less time to complete reflex cytology than women in the earlier period. This shorter observation window may therefore have led to an underestimation of true reflex cytology participation. When adjusting for the decrease in reflex cytology on overall participation, rates remain unaffected with adjusted participation rates of 49.3% after the new self-sampling policy compared to 42.0% for the control group (data not shown). Therefore, the decrease in reflex cytology participation does not, however, impact the overall screening participation rate, indicating that the broader implementation of self-sampling has resulted in a net increase in the overall screening participation. The additional step in the process for hrHPV-positive self-sampling participants could potentially explain the observed dropout. Reducing barriers to cervical smear testing, particularly for those who have already taken the step to use self-sampling, could further improve participation rates and ultimately the overall effectiveness of the screening programme.

A randomised trial from the U.S. reported a 17-percentage-point increase in participation among under-screened participants and a 14-percentage-point increase among adherent participants following the direct mailing of self-sampling kits, compared to participants receiving usual care.^7^ Similarly, a recent meta-analysis by Costa et al., reported a 13.2-percentage-point increase in participation following the mailing self-sampling kits, compared to the standard approach of an invitation for a smear test.^12^ These participation gains are slightly higher than those observed in our study. These differences may be explained by the study design and population: The U.S. study and the meta-analysis only included randomised controlled trials, whereas our study was conducted within a nationwide, population-based screening programme. Additionally, the U.S. trial mailed kits to all participants in the intervention arm, whereas our study mailed kits only to 30-year-olds and to non-responders after 12 weeks.

In a Danish randomised controlled trial, nested within a region (the Central Denmark Region) of the Danish organised cervical cancer screening programme, kits mailed with the second reminder led to a higher participation rate (38.0%) compared to the second reminder for regular cytology screening alone (25.2%).^13^ This 13-percentage-point increase is higher than the 8-percentage-point increase observed in our study. This trial targeted a more reluctant group, whereas our study evaluated total participation across the programme. When recalculated for total participation, the Danish study reported rates of 74% in the directly mailed self-sampling group and 69% in the control group, reflecting a 5-percentage-point increase, lower than the increase observed in our study. Moreover, the Danish study was conducted in one region of the national programme, whereas our study represents a nationwide implementation, which may account for differences in participation rates and generalisability.

Additionally, a Czech study reported attendance rates of 13.4% with directly mailed self-sampling kits compared to 5.0% in the control group (invitation letter for a smear test).^14^ This 8% increase aligns with our findings, but the Czech study involved only 50–65-year-olds non-participants, making it less directly comparable to our broader study population.

The strength of our study is that it is performed in a national organised hrHPV-based cervical cancer screening programme, in contrast to other studies, which are mainly trials. As the first country worldwide to implement direct mailing of self-sampling kits on a nationwide scale, our study provides valuable insights into the real-world effectiveness of this strategy, demonstrating its potential to increase participation in the population. The large sample size and population-based approach also enhance the generalisability of our findings.

Our study has some limitations. We focused on participation rates, and did not analyse screening outcomes (i.e. HPV positivity, cytology, and histology results). This is because a new self-sampling test was introduced at the same time as the policy change, which could have affected these outcomes and complicated interpretation. While increasing participation is expected to improve the effectiveness of the cervical screening programme by enabling earlier detection of lesions, our data do not allow direct assessment of CIN2+ detection rates. Future studies with longer follow-up and consistent testing protocols are needed to evaluate the long-term clinical impact of the new self-sampling policy. Furthermore, we were unable to assess the reasons behind the initially lower participation in reflex cytology among hrHPV-positive self-sampling participants. It is possible that this participation rate will improve over time as more individuals complete follow-up testing. Future research should explore the underlying barriers to timely follow-up testing and identify ways to support and improve subsequent participation among those with a positive self-sampling result. A further limitation of this study is the relatively short follow-up period of 3–8.5 months, depending on the date of invitation. As the reminder is sent 12 weeks after the primary invitation, some late responders may not have been fully captured within our study window. However, this follow-up period corresponds to the standard annual cut-off date (March 31 of the following year) used in the national monitoring of the screening programme, ensuring consistency and comparability across the years. Although the short window may slightly underestimate absolute participation rates, routine monitoring data indicate that the vast majority of women who respond to either the invitation or the reminder do so within the first few months. Therefore, while extended follow-up might yield marginally higher participation rates, it is unlikely to substantially affect the relative differences we observed between 2022 and 2023. Additionally, although censoring was not explicitly incorporated into the analysis, the uniformity in the timing of invitations (data not shown) suggests that censoring is unlikely to have had a significant impact on the results. Nevertheless, future studies with longer follow-up may provide a more comprehensive understanding of the long-term effects of the new self-sampling policy. Another limitation of this study is the before-and-after design, which does not fully account for ongoing secular trends in self-sampling uptake. National monitoring data show only modest increases in uptake from 21% in 2020 to 25% in 2022.^2^ The sharp rise observed immediately after July 2023 is therefore unlikely to be explained by this gradual trend alone. Nonetheless, the absence of a parallel control group limits causal inference, and our findings should be interpreted in this context. Lastly, national monitoring data have demonstrated a gradual decrease in participation rates over recent years, although participation within a given year remains relatively stable. This downward trend could partly influence differences observed between the time periods examined. However, given that participation is generally declining, the observed increase associated with the introduction of self-sampling in our study likely represents a conservative estimate of its true effect, suggesting that the positive impact of the intervention may be even greater than our results indicate.

It is also unclear whether the observed increase in participation reflects a general preference for self-sampling among participants or the convenience of receiving a kit directly at home. Understanding participant preferences could help refine future screening policies. Previous studies have shown high acceptability of self-sampling kits,^15^ suggesting that both factors may play a role.

One additional consideration is the potential waste of self-sampling kits when kits are mailed to individuals who do not use them, or fail to complete the testing process. This challenge highlights the importance of balancing accessibility with efficient resource use. Evidence from previous studies suggests that opt-out strategies, such as directly mailing kits, are more effective in increasing participation compared to opt-in approaches.^16^ This trade-off should be carefully considered when implementing nationwide self-sampling initiatives within organised programmes.

Direct mailing of self-sampling kits may also have implications for the cost-effectiveness of cervical cancer screening. Several studies have shown that this approach can be more cost-effective, particularly when targeting non-responders, compared to traditional reminder letters.^17^^,^^18^ As demonstrated in our study, the increased use of self-sampling reduces the need for women to visit a GP for a hrHPV-test, thereby lowering costs associated with in-person consultations. While formal health-economic modelling was beyond the scope of the current study, such work has been undertaken by our group and accepted for publication.^19^ These modelling results provide detailed insights into programme efficiency, sustainability, and programme cost-effectiveness, and will be important to inform future policy decisions.

In conclusion, the implementation of directly mailing self-sampling kits within a national screening programme is recommended to enhance participation and potentially improve the overall effectiveness of cervical cancer screening programmes.

## Contributors

EMGO was involved in performing the data analyses, visualisation of the results and writing the draft of the article. AGS was involved in the data collection. IMCMdK was responsible for the supervision of the project. All authors have accessed and verified the underlying data reported in this manuscript. All authors were involved in the conceptualisation of the study and reviewed and approved the final article for publication.

## Data sharing statement

Data sources and handling of the dataset used in this study are described in the Methods. Further details and other data that support the findings of this study are available from the corresponding authors upon request.

## Ethics statement

Not applicable.

## Declaration of interests

The authors declare no conflicts of interest.
